# Immune Checkpoint LAG3 and Its Ligand FGL1 in Cancer

**DOI:** 10.3389/fimmu.2021.785091

**Published:** 2022-01-17

**Authors:** An-Ping Shi, Xi-Yang Tang, Yan-Lu Xiong, Kai-Fu Zheng, Yu-Jian Liu, Xian-Gui Shi, Yao Lv, Tao Jiang, Nan Ma, Jin-Bo Zhao

**Affiliations:** ^1^ Department of Radiology & Functional and Molecular Imaging Key Lab of Shaanxi Province, Tangdu Hospital, Fourth Military Medical University (Air Force Medical University), Xi’an, China; ^2^ Department of Thoracic Surgery, Tangdu Hospital, Air Force Medical University, Xi’an, China; ^3^ College of Basic Medicine, Air Force Medical University, Xi’an, China; ^4^ Department of Ophthalmology, Tangdu Hospital, Air Force Medical University, Xi’an, China

**Keywords:** LAG3, FGL1, immune checkpoint, immune therapy, immune response, tumor

## Abstract

LAG3 is the most promising immune checkpoint next to PD-1 and CTLA-4. High LAG3 and FGL1 expression boosts tumor growth by inhibiting the immune microenvironment. This review comprises four sections presenting the structure/expression, interaction, biological effects, and clinical application of LAG3/FGL1. D1 and D2 of LAG3 and FD of FGL1 are the LAG3-FGL1 interaction domains. LAG3 accumulates on the surface of lymphocytes in various tumors, but is also found in the cytoplasm in non-small cell lung cancer (NSCLC) cells. FGL1 is found in the cytoplasm in NSCLC cells and on the surface of breast cancer cells. The LAG3-FGL1 interaction mechanism remains unclear, and the intracellular signals require elucidation. LAG3/FGL1 activity is associated with immune cell infiltration, proliferation, and secretion. Cytokine production is enhanced when LAG3/FGL1 are co-expressed with PD-1. IMP321 and relatlimab are promising monoclonal antibodies targeting LAG3 in melanoma. The clinical use of anti-FGL1 antibodies has not been reported. Finally, high FGL1 and LAG3 expression induces EGFR-TKI and gefitinib resistance, and anti-PD-1 therapy resistance, respectively. We present a comprehensive overview of the role of LAG3/FGL1 in cancer, suggesting novel anti-tumor therapy strategies.

## Introduction

Tumors are a major public health concern and, currently, immunotherapy is the most promising tumor treatment (1). The targets of immunotherapy are immune checkpoints, which are immune cell or tumor cell receptors, exerting positive or negative regulation of the immune system. Seventeen immune checkpoints can be divided into two groups due to their roles in the immune microenvironment, immune activating checkpoints, and immune inhibitory checkpoints (2). Lymphocyte-activation gene 3 (LAG3, CD223) is an immune inhibitory checkpoint and is expressed on the surface of lymphocytes (3, 4), such as CD4^+^ T cells, CD8^+^ T cells, natural killer (NK) cells, NK T (NKT) cells, and regulatory T (Treg) cells (5, 6), as well as stored in the lysosomes, which appear on the surface faster when T cells are activated (7). LAG3 inhibits the tumor immune microenvironment by accelerating T cell exhaustion and blocking T cell proliferation (8). Major histocompatibility complex (MHC) II is a canonical ligand of LAG3, which is irrelevant with LAG3-mediated T cells dysfunction (9), MHC-II may interact with D1 domain of LAG3 but more evidence are warranted for further protein-protein interaction (10). There are several other LAG3 ligands, including lymph node sinusoidal endothelial cell C-type lectin (LSECtin), Galectin-3, α-synuclein, fibrinogen-like protein 1 (FGL1) also called fibrinogen-associated protein 1 (FREP1), HRFREP-1 or hepassocin (11–14), and FGL2, belonging to the FREP family, which inhibits T cell activation by binding with LAG3. However, the intracellular signaling pathways of LAG3 and FGL1, both of which play a role in the regulation of immune cell function, cytokine production, and tumor growth, remain unknown. Progress has been recently achieved in immunotherapy targeting LAG3 and FGL1 and LAG3/FGL1 expression has been associated with therapeutic effect prediction and prognosis, as well as therapy resistance. In this review, we summarize the structure, expression, interaction, biological effect, and relevant clinical research on LAG3 and FGL1, to further explore the therapeutic potential of this pair of immune checkpoints and effectively apply them in clinical treatment. In tumors, such as kidney renal clear cell carcinoma (KIRC), non-small cell lung cancer (NSCLC), colorectal cancer, hepatocellular carcinoma (HCC), primary central nervous system lymphoma (PCNSL), diffuse large B-cell lymphoma (DLBCL), and muscle invasive bladder cancer (MIBC), higher expression of LAG3 indicates a poor prognosis. However, in some tumors, such as gastric cancer and melanoma, a higher expression of LAG3 indicates a better prognosis.

## LAG3/FGL1 Structure and Expression

### LAG3/FGL1 Structure

**Figure 1:**
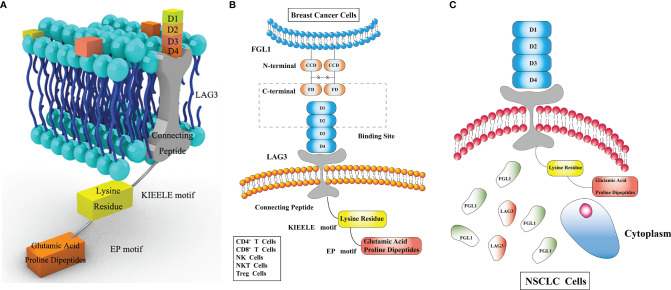
**(A)** Lymphocyte-activation gene 3 (LAG3) consists of three regions. The extracellular region consists of four immunoglobulin superfamily (IgSF) domains, D1, D2, D3, D4. The transmembrane region consists of a long connecting peptide connected with D4. The intracellular region includes a serine phosphorylation site, a “KIEELE” motif and an “EP” motif. The “KIEELE” motif consists of a lysine residue, and glutamic acid and proline dipeptides constitute the main part of the “EP” motif. The “EP” and “KIEELE” motifs are associated with T cell proliferation and activation. **(B)** Fibrinogen like 1 (FGL1) consists of an N-terminal signal recognition peptide (coil-coil domain, CCD) and a C-terminal fibrinogen-like domain (FD), without any membrane-crossing region. FD in FGL1, and D1 and D2 in LAG3, are the LAG3 and FGL1 interaction sites. LAG3 is expressed on the surface of CD4^+^ T cells, CD8^+^ T cells, natural killer (NK) cells, NK T (NKT) cells and regulatory T (Treg) cells. **(C)** FGL1 is expressed in various tumors. It is found on the surface of breast cancer cells but in the cytoplasm in non-small cell lung cancer (NSCLC) cell lines.

*LAG3*, residing on chromosome 12 (12p13.32), encodes a type I transmembrane protein, which is a member of the Ig superfamily (Ig SF) and consists of three regions: the extracellular, transmembrane, and intracellular regions (15). The LAG3 gene contains eight exons and the mature LAG3 protein contains 470 aa. The extracellular region consists of four immunoglobulin superfamily (IgSF) domains, D1, D2, D3, and D4, which include eight cysteine residues and 4 N-linked glycosylation sites. D1 belongs to V-SET domain whereas D2/3/4 belong to C2-SET domain, which is encoded by exons II/III/IV/V/VI. The transmembrane region, a long connecting peptide, is connected with D4, which is encoded by exon VII. The intracellular region includes a serine phosphorylation site, a “KIEELE” motif and an “EP” motif. The “KIEELE” motif contains a lysine residue and can inhibit T cell activation (16). The “EP” motif mainly consists of glutamic acid and proline dipeptides (17)**(** **Figure 1** **)**. The intracellular domain of LAG3 is associated with T cell proliferation and cytolytic function (4), however, the function of the intracellular region remains unclear. In addition, the hydrophobic leader peptide is encoded by exons I and II, and the highly charged cytoplasmic region is encoded by exon VIII (18).

Fibrinogen consists of five regions, including the central nodule, coiled-coil domain, α/β/γ C-terminus domains, of which the β and γ domains are highly homologous with fibrinogen-like globe (FBG) domains (19). FGL1 is a member of the FREP family, and contains two 34 kda homodimers connected by disulfide bonds, forming a 68 kda protein. FGL1 consists of an N-terminal signal recognition peptide (coil-coil domain, CCD) and C-terminal fibrinogen-like domain (FD), which binds LAG3, without any membrane-crossing region (20, 21).

### LAG3/FGL1 Expression

LAG3 is expressed on the surface of lymphocytes (4), including CD4^+^ T cells, CD8^+^ T cells, NK cells, NKT cells, and Treg cells, LAG3 is also colocalized with CD4 in recycling endosomes, secretory lysosomes, microtubule organizing center (22), which appear on the surface faster to inhibit the function of T cells when T cells are activated, and their translocation is regulated by the protein kinase C (PKC) signaling pathway (7). However, Ma et al. found that LAG3 is not only expressed on lymphocytes but also in NSCLC cell lines, including H226, H1299, A549, and BEAS-2B cells. Furthermore, LAG3 was also found in the cytoplasm (23), further research is required to verify this. LAG3 is expressed in various tumors, such as KIRC, gastric cancer, breast cancer, B-cell lymphoma, and lung cancer (3, 24–26). In gastric cancer, compared to CD68^+^ macrophages, higher expression of LAG3 can be found in CD3^+^/CD8^+^ T cells (27). The differential expression of LAG3 in different tumor cells indicates a different patient prognosis. This will be discussed in detail in Section *Prognostic Markers*.

LAG3 expression is correlated with that of other immune checkpoints. In gastric cancer, the expression of programmed cell death protein 1 (PD-1) is positively correlated with that of LAG3 and T-cell immunoglobulin domain and mucin domain 3 (TIM3), whereas the expression of LAG3 is associated with the expression of TIM3 (27). The co-expression of LAG3 and PD1 or PD ligand 1 (PD-L1) has been proven in breast cancer. LAG3 and PD1 double positive expression is evident either in triple negative breast cancer (TNBC), or in estrogen receptor^+^/progesterone receptor ^+^(ER^+^/PR^+^ breast cancer (28). Moreover, in TNBC, a higher LAG3 expression is related to PD-L1 expression (29). Wu et al. obtained similar results showing that half of PD-L1^+^ cases exhibited LAG-3 co-expression (30). In breast cancer, Liu et al. found that the synergistic effects of LAG3 with other immune checkpoints, including PD-L1, TIGIT, CD27/28/40/48/86, cytotoxic T lymphocyte-associated antigen-4 (CTLA-4), ICOS, and IDO1, which may co-regulate the immune microenvironment of breast cancer, are prominent (31). In ovarian cancer, interleukin (IL)6/10 and tumor-associated antigen-presenting cells (APCs) can significantly promote the co-expression of LAG3 and PD1 on the surface of CD8^+^ tumor-infiltrating lymphocytes (32). In gastric cancer, the inhibitory ligands of LAG3, LSECtin, and MHC II are correlated with the expression of PD-L1, which may be indicative of the OS of patients with gastric cancer (33). The dual or triple positive expression of immune checkpoints may affect tumor prognosis or treatment. This will be discussed in detail in Section 4.

LAG3 expression can be affected by various factors. In KIRC, the expression of LAG3 mRNA and tumor-intrinsic protein are associated with methylation regulation. In particular, the upregulation of LAG3 mRNA may be related to LAG3 promoter hypomethylation, as well as the methylation of LAG3 downstream genes. This upregulation is especially evident in CpG site 15 (transcriptional repressor of the CTCF binding site). However, the hypomethylation of CpG site 4 and the methylation of CpG site 8, both of which are associated with LAG3 protein expression, strongly inhibit LAG3 mRNA expression (34). In melanoma, LAG3 mRNA expression is associated with LAG3 promoter methylation. LAG3 methylation is negatively associated with LAG3 mRNA expression. However, the methylation of bead 14, in the gene body, and bead 15, in the CTCF binding site, is significantly positively related to LAG3 mRNA expression (35). The degradation of LAG3 is regulated by disintegrin and metalloproteinase domain-containing protein 10/17 (ADAM10/17), TNF-α, TIM3, CD62L, and VEGFR2 (36). The activity of ADAM17 is regulated by TCR- and PKCθ-dependent serine phosphorylation (37). The expression of LAG3 can also be affected by various cytokines. The increased levels of IL-10 and IL2/12 from activated T cells can effectively upregulate the expression of LAG3 (38). Furthermore, LAG3 expression is induced by IL-7/15/27 (14, 39), whereas IL-4 can effectively downregulate the expression of LAG3 (40). Moreover, higher LAG3 protein expression is associated with an increase in the levels of IFN-γ (41). Thus, LAG3 expression is affected by several factors, especially by epigenetic regulation. However, LAG3 regulation at the gene, transcription, post-transcription, translation, and post-translation levels warrants further research.

FGL1, initially found in hepatocytes, plays a role in metabolism (42). In recent years, high FGL1 expression was found in tumor cells (21), but conflicting results have been reported regarding the specific expression site. Chen et al. found that FGL1 is mainly expressed in the cytoplasm of NSCLC tumor cells using immunofluorescence staining (43), whereas Du et al. showed that FGL1 accumulated on the surface of breast cancer cells (28), this discrepancy can be attributed to that FGL1 may server as an oncogene in NSCLC in addition to its immune checkpoint role (44). FGL1 is a LAG3 ligand. Nevertheless, the specific immunological mechanism underlying the function of FGL1 warrants further research.

Thus, although LAG3 is primarily expressed on lymphocytes it is also found in NSCLC tumor cell lines and its expression is regulated by various factors. As the majority of studies have focused on LAG3 and fewer on FGL1, the expression sites of FGL1 remain controversial, and its regulation remains unknown. Hence, this topic shows enormous potential in tumor research.

## The Interaction Between LAG3 and FGL1

Using genome-scale receptor array and flow cytometry, Wang et al. proved that FGL1 is a LAG3 ligand, and that the interaction between them is conserved, stable, and specific, both in humans and mice. The LAG3 and FGL1 interaction sites are the D1 and D2 of LAG3 and the FD of FGL1. The interaction of LAG3 and FGL1 may cause changes in the tumor immune microenvironment, such as the reduction of IL-2 levels (45). The interaction site of FGL1 and LAG3 is different from that between MHC-II and LAG3. The Y73F mutation in mice and Y77F mutation in humans can effectively inhibit the binding of LAG3 to MHC-II without influencing the binding between FGL1 and LAG3. Thus, mAb C9B7W (anti-LAG3) can specifically inhibit the interaction between LAG3 and FGL1, but further research is required to prove this (14). Studies on the interaction mechanisms between LAG3 and FGL1 in HCC showed that oxysophocarpine promotes the therapeutic effect of the anti-LAG3 mAb by inhibiting the expression of its ligand FGL1 *via* blocking the IL-6 related JAK2/STAT3 signal pathway (46).

As we showed before, FGL1 is the major LAG3 ligand. However, according to the IHC results in the abovementioned studies, FGL1 is expressed on the surface of breast cancer cells, or in the cytoplasm of NSCLC cells, whereas LAG3 is expressed on the surface of lymphocytes. Hence, how FGL1 in the cytoplasm can interact with LAG3 on the cell membrane and if second messengers are involved in this process remain to be determined. Furthermore, LAG3 inhibits the proliferation and cytokine production induced by the TCR complex (47, 48). However, the changes in the relevant intracellular signaling pathway remain unknown, as well as the changes occurring after FGL1 binding to LAG3. Therefore, the potential to explore the interactive mechanisms and related signal pathway changes is enormous, which may further elucidate the inhibitory effect of LAG3/FGL1 on tumors.

## The Biological Effect of LAG3/FGL1 on Different Tumors

### Immune Cell Function

LAG3 regulation is tightly associated with immune cell infiltration but differs in various tumors. In some tumors like breast cancer, lung adenocarcinoma, and lung squamous carcinoma, the high expression of LAG3 is tightly associated with the increased infiltration of immune cells, including T cells, B cells, cytotoxic lymphocytes, especially that of NK cells and DC cells (31, 49). However, Du et al. demonstrated that in breast cancer, the proliferative and secretory function of T cells can be significantly decreased by the double positive expression of LAG3 and PD1. Moreover, the amount of LAG3^+^PD1^+^ T cells is different in various molecular subtypes of breast cancer, with the highest being in TNBC and lowest in ER^+^/PR^+^ breast cancer (8, 28). From above all, the discrepancy of LAG3 exerts positively or negatively in immune functions can be attributed to the complexity of tumor immune microenvironment and tumor heterogeneity, besides, LAG3 may not create immunosuppression when acting alone, but only when acting in combination with other immune checkpoints such as PD1. Some studies suggested that pHLA-II is a LAG3 receptor (50–52), and recently, Maclachlan et al. showed that LAG3 can directly and competitively bind with pHLA-IIto influence the bind of CD4 with pHLA-II, further inhibiting T cell activation (53).

The role of LAG3 in tumor immune can be modulated by methylation. In KIRC, hypomethylation of the LAG3 promoter and hypermethylation of CpG site 15 are related to increased immune cell infiltration (34). In melanoma, the hypomethylation of beads 1 to 13 is associated with higher LAG3 mRNA expression. The high LAG3 mRNA expression and the hypermethylation of beads 14 and 15 are positively related to lymphocyte score and leukocyte fraction. Furthermore, the hypomethylation of LAG3 promoter and the hypermethylation of the CTCF binding cite may increase the infiltration of immune cells (35), indicating that the methylation of LAG3 may become a new epigenetic marker for tumor immune cell infiltration.

### Cytokine Production

The regulation of LAG3 is tightly associated with the production of cytokines, especially immune-related cytokines. LAG3 can stimulate the production of mature DC-derived IL-12 and TNF-α (54). In melanoma, the methylation of LAG3 promoter regions may inhibit the mRNA expression of IFN-γ-and IFN-γ-regulated genes, including STAT1/2, JAK2, and IRF9 (35). In KIRC, the mRNA expression of STAT1/2, JAK2, and IRF9 is promoted due to the hypomethylation of the LAG3 promoter and the hypermethylation of CpG sites 14/15, which is associated with an increase in IFN-γ production (34). In ovarian cancer, compared to PD1^+^LAG3^–^ or LAG3^–^PD1^–^ tumor-infiltrating lymphocytes, the triple positive expression of CD8, LAG3, and PD1 in lymphocytes can potently inhibit the production of TNF-α, IFN-γ, and IL-2 (32). In follicular lymphoma, the co-expression of LAG3 and PD-1 on T cells induces T cell exhaustion and inhibits the production of cytokines, such as IFN-γ and IL-2 (55). In MIBC, the high expression of LAG3 may induce the secretion of more inhibitory cytokines, including IL-10 and TGF-β, which inhibit tumor contexture and promote tumor immune escape (56).

The regulation of FGL1 is tightly associated with cytokine production. In the 3A9 T cell line, in which LAG-3 overexpression is induced by IL-2, the suppressive effect of FGL1 is more evident on T cell proliferation, whereas the use of anti-FGL1 mAb leads to a positive regulation of TNF-α and increased IFN-γ levels, which can restore the activation of T cells (43).

### Tumor Growth, Invasion, and Migration

FGL1 may simultaneously serve as oncogene and tumor suppresser gene in various cancers. The downregulation of FGL1 inhibit the growth of MC38 colon cancer cells (43) and SGC-7901 gastric cancer cell (44), especially, in FGL1-KO MC38 colon cancer mouse model, the administration of anti-FGL1 and anti-LAG3 mAbs shows favorable anti-tumor effect, this effect anti-tumor effect is eliminated when anti-CD8^+^ and anti-CD4^+^ mAbs are simultaneously used, which is in line with the results in Rag1-KO C57BL/6 mice (T/B cells deficiency); besides, the downregulation of FGL1 may involve in EMT (epithelial-mesenchymal transition) to inhibit the invasion, proliferation, and migration of SGC-7901 gastric cancer cell. However, the downregulation of FGL1 promote the growth of LKB1 mutant lung cancer cells and HCC cells, in the LKB1-overexpressing A549 lung carcinoma cell line, FGL1 silencing leads to an increased cell growth rate and to increased cell migration and promotes tumor angiogenesis and EMT (57); in FGL1-null mice, the growth rate of HCC is faster compared to that in wild type mice, and that the expression of FGL1 is inhibited during HCC development, which may be attributed to the activation of Akt and mTOR (21).

The role of FGL1 differs in these tumors can be attributed to the tumor heterogeneity, the results of FGL1 in MC38 colon cancer cells are mor complete and compelling, it is hard to explain the results of the downregulation of FGL1 regulates EMT in SGC-7901 gastric cancer cell and LKB1-overexpressing A549 lung carcinoma cell, which are totally reverse and incredible, it warrants more exploration.

## Clinical Application of LAG3/FGL

### Treatment and Curative Effect

Many mAbs target LAG3, most of which are undergoing clinical trials, including IMP321(first LAG3 fusion protein) and relatlimab (first LAG3 mAb), and show good potential in tumor immunotherapy (58). There are only four clinical trials targeting at LAG3 have completed the recruitment, all of them are in phase 1 and aim at testing the safety and tolerance of IMP321 and Sym022. The structure of IMP321 is similar to that of LAG3, with D1-D4 domains to activate monocytes, DCs, and tumor-specific T cell immune responses by competitively binding to MHC-II (59). It has proved that IMP321 can inhibit tumor growth and prolong the PFS of advanced renal cell carcinoma patients when dose >6mg, which can also significantly activate CD8^+^T cells (NCT00351949) (60). The other two completed trials have not reported. The first clinical trial to evaluate the safety and tolerance of Sym022 has completed and irAEs comes to occur when dose >3mg/kg Q2W (NCT03489369). Other recruiting anti-LAG3 clinical trials are mainly in phase 1 to evaluate the application of LAG3 related monotherapy and combination therapy in various tumors **(** **Supplementary Table 1** **)**. There are currently no mAbs targeting FGL1.

Monotherapy targeting LAG3 is emerging in a variety of tumors. In chronic lymphocytic leukemia (CLL), immunotherapy, such as anti-PD1/CTLA-4 immunotherapy, shows little benefits in patient survival, and the higher expression of LAG3 in CLL contributes to tumor immune escape, as well as indicates poor prognosis. However, the brand new mAb targeting LAG3 relatlimab (BMS 986016) shows great antitumor effect in CLL by restoring the responses of NK cells and T cells. When combined with relatlimab and lenalidomide (immunomodulatory drug), the production of IL-2 and NK cell-mediated ADCC are enhanced (61). Zhai et al. developed a cyclic peptide named C25, which shows a significant anti-tumor effect in CT26 (colorectal cancer), B16, and B16-OVA (melanoma) mouse tumor models by inhibiting the interaction between LAG3 and MHC-II, as well as the increased CD8^+^ T cell and decreased Treg cell levels. Furthermore, C25, which is considered as an alternative for tumor immunotherapy, can effectively increase the production of IFN-γ, indicating its prominent anti-tumor effect (62). In MIBC, low expression of LAG3 results in high levels of FGFR3 gene mutation, which may be sensitive to FGFR3-targeted treatment (56). In advanced gastric cancer, compared to low expression of LAG3, high expression of LAG3 and OX40 indicates better therapeutic effect of nivolumab. The HR of progression-free survival (PFS) in the high expression group was 0.1164 and 0.0926 for CD4^+^/CD8^+^ and LAG3^+^ T cells, respectively, indicating that the high expression of LAG3 is a protective factor and predicts the therapeutic effect of nivolumab (63). The nanobody can noninvasively detect the upregulation of LAG3 following anti-PD-1 treatment, which may be used for therapeutic effect prediction, but its efficiency warrants further research (64). In HCC HepG2 and Hepa1-6 cell lines, oxysophocarpine significantly inhibits cell proliferation and migration, and promotes apoptosis. Oxysophocarpine also enhances the therapeutic effect of anti-LAG3 mAb in HCC, promoting the function and cytotoxicity of tumor-related CD8^+^ T cells. The underlying mechanism involves the inhibition of IL-6-related JAK2/STAT3 signaling pathways, which can distinctly inhibit the expression of FGL1 (46).

Combination therapy may have a better treatment effect on tumors. In a phase I/II study, in patients with advanced melanoma who failed to respond to anti-PD-1 therapy, the combination therapy of relatlimab and nivolumab showed a satisfying tolerance with an objective response rate (ORR) of 11.5% (65).The combination of anti-LAG3 and anti-PI3Kδ antibodies has a therapeutic potential to restrict tumor burden, and the results show that the anti-LAG3 mAb exerts its effects within tumors (66, 67). Compared to the single positive group, the double block of LAG3 and PD1 can enhance the synergistic effect by more than 50-fold (68). Peptide and DNA vaccines that double block PD-1 and LAG3 showed a great anti-tumor effect, especially in a MycCaP prostate cancer model, which significantly increased the levels of CD8^+^T cells, which express more immune checkpoints, underlying the role of APCs (69). It is urgent to develop an effective treatment for malignant pleural mesothelioma, and combination immunotherapy has received increasing attention recently. In the mouse AB1-HA BALB/cJ mesothelioma model, compared to the control group, blocking both PD-L1 (durvalumab) and LAG3 effectively inhibited tumor growth and could be beneficial for mouse survival (70). In the mouse MC38 colorectal cancer model and A375 melanoma model with PD-L1 knock in, the use of IBI323 (mAb double blocking PD-L1 and LAG3) showed a superior anti-tumor effect by inhibiting the interaction between PD-1/PD-L1 and LAG3/MHC-II, and enhanced the activity of immune stimulation, increasing the amount of CD4^+^ and CD8^+^ T cells (71). In the MC38 and CT26 colorectal cancer tumor mouse model, FS118 (bispecific antibody blocking PD-L1 and LAG3) showed a favorable anti-tumor effect by restoring the activation of T cells and was well tolerated without any toxicity (72). The combination of anti-PD-1 and anti-LAG3 therapy, which regulates the populations of T cells, may be beneficial in Hodgkin lymphoma (HL). Nagasaki et al. showed that LAG3 inhibits the anti-tumor effect of anti-PD-1 and anti-LAG3 therapy in HL by inhibiting the CD4+ T cell responses (73), and that chemotherapy with ABVD (doxorubicin, bleomycin, vinblastine, and dacarbazine) shows little therapeutic effect with higher infiltration of LAG3^+^TILs. The combination immunotherapy with anti-PD-1/LAG3 distinctly increases the MHC-II responses. Compared to its expression in DLBCL, the expression of LAG3 is three-fold higher in HL, and the expression level of LAG3 in primary mediastinal B-cell lymphoma is similar to that in HL (74). In DLBCL, the expression of LAG3 is the highest, and is associated with the expression of PD-L1 and tumor mutation burden, and the double block of LAG3 and TIM3 restores the function of anti-DLBCL T cells. However, further preclinical and clinical trials are required to verify the therapeutic effect of anti-LAG3/TIM3 antibodies (75). Peng et al. found that the expression of LAG3 after neoadjuvant radiotherapy (NRT) significantly increased in rectal cancer, especially for short-term NRT, and that this high expression may induce a better treatment effect. Thus, it is reasonable to consider that the combination of NRT with immunotherapy may be an alternative for patients with advanced rectal cancer (76).

Except for the monotherapies and combination therapies mentioned above, the gene editing technology CRISPR-Cas9 points out a new direction to enhance the efficacy of tumor immunotherapy, as knocking out PD-1 and LAG3 of CAR-T cells can significantly enhance the anti-tumor effect and inhibit the immunosuppressive microenvironment (77, 78). The combination therapy may bring more anti-tumor effects than single agent therapy, but also more immune-related adverse effects (irAEs), like immune dermatitis (47%-65%), colitis (30-48%) and hepatitis (5%-30%) (79, 80). Some intersections between tumor immune and autoimmune functions lead to the occurrence of irAEs. In particular, T helper (Th) 1 and Th17 cells play a role in the irAEs of tumor immunotherapy, especially the protein podoplanin on Th17 cells, the depletion of which may induce auto-immune disease by enhancing the infiltration of T cells, as well as inhibit tumor growth (81). Furthermore, the protein C receptor and glycosphingolipid receptor affect the regulation of Th17 cells, which may simultaneously lead to anti-tumor effect and excessive autoimmunity (82, 83). Thus, enhancing the function of tumor-specific T cells rather than that of other T cell subtypes is the future direction in tumor immunotherapy (84).

### Prognostic Markers

The LAG3 expression level can be a marker of poor prognosis in various tumors. In NSCLC and advanced CRC, the high expression of FGL1 and LAG3 are related to the poor 5-year OS, respectively (43, 85), high level of soluble LAG3(>377pg/ml) predicts unfavorable PFS and OS in HNSCC (86). More LAG3^+^ cells induce the immunosuppressive microenvironment and predict the poor prognosis, in EBV-positive and MLH1-defective gastric cancer, high infiltration of LAG3^+^ cells may induce immune escape in tumors with fewer IFN-γ^+^ cells and perforin-1^+^ cells and more Treg cells and M2 macrophages in this subtype of gastric cancer (87), similar results can be found in MIBC and pancreatic ductal adenocarcinoma (56, 88). The co-expression of FGL1 and LAG3 are negatively correlated with PD-L1 expression and CD8^+^T cell number in HCC (89), the co-expression of LAG3 and TIM3 may serve as a T cell exhaustion marker in PCNSL (90, 91), both of the co-expression predict poor survival

LAG3 and its ligands are associated with the prognosis of gastric cancer. The single expression of LAG3^+^ and the dual expression of PD-1^+^/LAG3^+^ predict better PFS, and the dual expression of TIM3^+^/LAG3^+^ predict better OS and PFS. Furthermore, in advanced gastric cancer, a higher ratio of LAG3^+^CD4^+^/CD4^+^ T cells and LAG3^+^CD8^+^/CD8^+^ T cells is associated with better prognosis, although, at the invasive tumor margin, higher LAG3 expression is associated with better prognosis (27). In FGL1^+^ gastric cancer, where a higher expression of FGL1 is positively associated with gastric cancer stage and lymph node metastasis, as well as the poor OS (44). In addition, MHC II and LSECtin, both are the ligands of LAG3, indicating a favorable survival in gastric cancer, and predict the treatment response to combination therapy with anti-PD1 and anti-LAG3 (33).

The prognostic value of LAG3 in breast cancer is controversial. LAG3 is associated with breast cancer stage, tumor size, tumor grade, and ER/PR/HER2 status. In particular, higher expression of LAG3 is found in stage I breast cancer (31), predicting the favorable survival in patients with ER^-^ and ER+ breast cancer (65, 92, 93), and longer OS and RFS are associated with higher LAG3 expression in TNBC (29). Compared to the “cold” immune subtype (immune desert subtype), the double positive expression of LAG3 and PD1 is tightly associated with tumor brain metastasis in the “hot” subtype (inflamed immune subtype). Further, Sobottka et al. found that the double positive expression of LAG3 and PD1 predicts negative prognosis in breast cancer patients, influencing the decreased DFS, especially for patients with metastasis (94).

The role of LAG3/FGL1 in prognosis prediction is controversial. LAG3 is considered a favorable prognostic marker, which may be attributed to the fact that a temporary high LAG3 expression after immunotherapy is associated with T cell activation and infiltration, leading to a better response to immune checkpoint inhibitors (5). The results that the co-expression predict diverse prognosis can be attributed to the interactions between LAG3 and other molecules are different; the discrepancy in breast cancer can be attributed to the various immune microenvironment of breast cancer subtypes. All above results have been folded in the **Supplementary Table 2**.

### Therapy Resistance

LAG3/FGL1 may be involved in tumor immune resistance. In metastatic NSCLC and melanoma, characterized by higher plasma FGL1 levels, poor outcome is found following anti-PD-1 therapy, indicating that FGL1 may play a role in tumor immune resistance (43). Furthermore, some patients with NSCLC may benefit from immunotherapy but fail to respond to EGFR-TKI treatment. Using multiplexed fluorescent immunohistochemical staining of pre- and post-EGFR-TKI therapy samples, higher LAG3 expression was found following EGFR-TKI treatment, which may contribute to the failure of EGFR-TKI therapy due to the negative regulation of LAG3 in the immune microenvironment. Furthermore, higher FGL1 expression may induce resistance to gefitinib, and FGL1 knockdown using siRNA inhibits cell viability, promotes apoptosis, and reduces IC50 values. The specific mechanism of apoptosis induction in gefitinib resistance may involve the inhibition of poly(ADP-Ribose) polymerase 1 and caspase3 expression (95, 96). The opposite results were reported in HCC, where the loss of FGL1 was found to induce therapy resistance. By performing functional studies in six HCC cell lines, higher FGL1 expression was associated with better response to sorafenib. Furthermore, the colony forming function and IC50 values were two- to three-fold higher in the low FGL1 expression group compared to those of the high FGL1 expression group. The specific mechanisms may involve the high expression of FGL1, which may influence MAPK and autophagy-related signaling, verified by FGL1 silencing (97). Anti-PD1/CTLA-4 therapy is an effective immunotherapy for the highly metastatic uveal melanoma. Durante et al. reported the role of LAG3 in anti-PD1 immunotherapy, where it acts as an exhaustion marker and its expression may contribute to the failure of anti-PD1/CTLA4 treatment (98).

## Perspective

**Figure 2:**
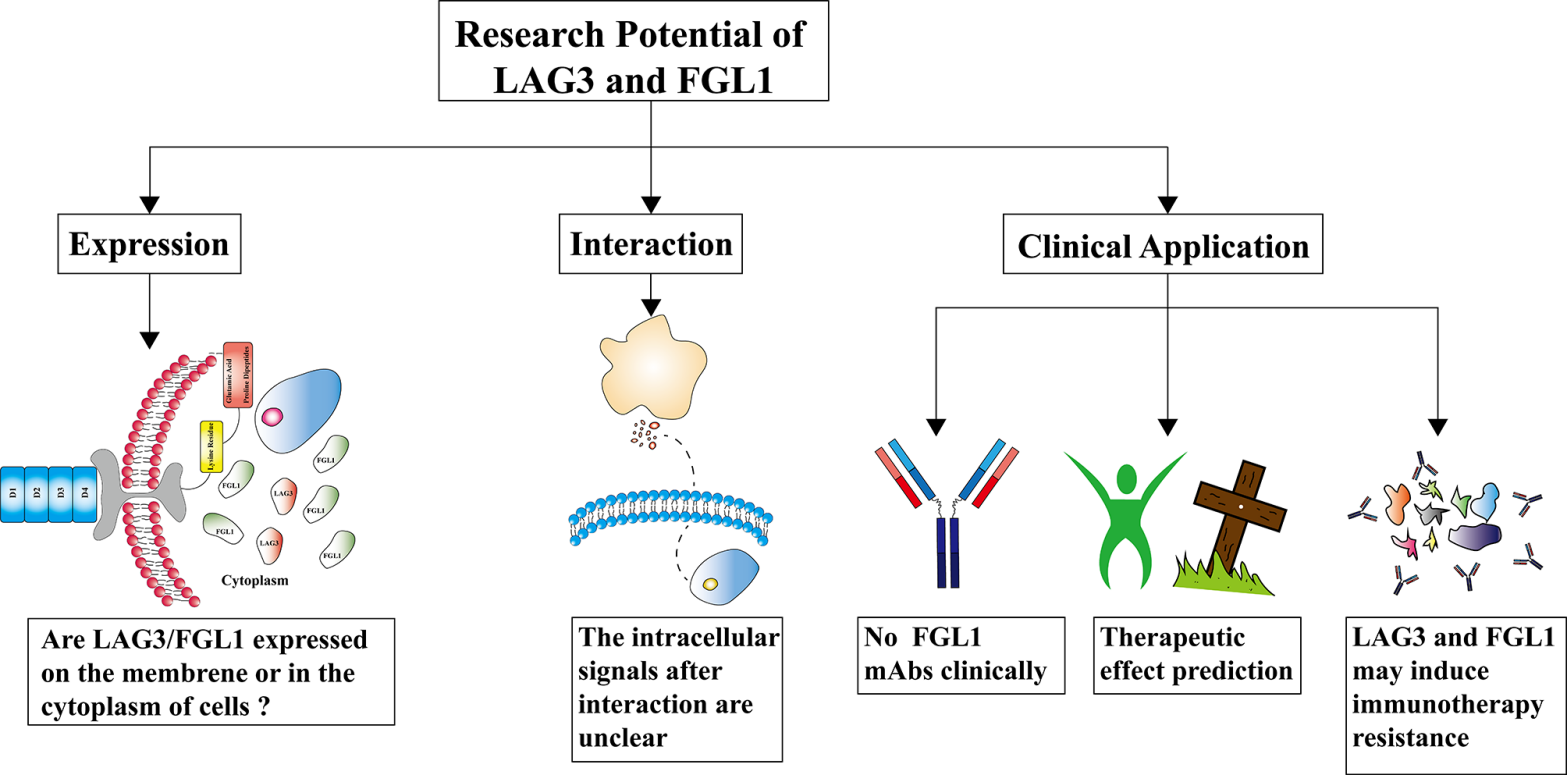
The research potential of lymphocyte-activation gene 3/fibrinogen like 1 (LAG3/FGL1). The expression of LAG3/FGL1 is controversial in tumors, especially that of FGL1. The mechanisms underlying the LAG3/FGL1 interaction remain unknown. How LAG3/FGL1 in the cytoplasm of NSCLC cell lines interact is unclear, and the relevant intracellular signaling changes warrant further research. Monoclonal antibodies (mAbs) targeting LAG3 are undergoing clinical trials and there are no clinical reports of FGL1 mAbs. The influence of LAG3/FGL1 expression in predicting the therapeutic effect varies in different tumors and lacks the underlying rationale. LAG3 and FGL1 may induce targeted therapy resistance, but if they induce immunotherapy resistance is currently unknown.

This review is mainly divided into four sections, discussing the structure/expression, interaction, biological effect, and clinical application of LAG3/FGL1, some parts of them show an enormous research potential for LAG3/FGL1 **(** **Figure 2** **)**.

LAG3 is mainly expressed on the surface of lymphocytes in a variety of tumors, exerting an inhibitory effect on immunity by binding with its ligands. However, it can also be expressed in the cytoplasm of NSCLC cell lines, but this needs further verification. Compared to LAG3, the expression of FGL1 has rarely been studied. Chen et al. proposed that in NSCLC, FGL1 is mainly accumulated in the cytoplasm, but Du et al. suggested that FGL1 is mainly expressed on the membrane of breast cancer cells. Thus, the expression site of FGL1 is controversial, and related to the use of specific interaction detection methods. The intracellular signaling pathways activated by LAG3/FGL1 remain unclear. How FGL1 in the cytoplasm can interact with LAG3 on the cell membrane and if second messengers are involved in this process remain to be determined. Therefore, studies focusing on the interaction between LAG3 and FGL1, as well as the related intracellular signaling pathways, may elucidate the specific mechanisms underlying the function of LAG3/FGL1 in immunity inhibition, and provide novel immunotherapy targets. Among the ongoing clinical trials of mAbs targeting LAG3, IMP321 and relatlimab trials have progressed rapidly in recent years, showing a favorable anti-tumor effect in melanoma. Most combination therapies involve the combination of anti-LAG3 and anti-PD-1 therapies, which can significantly enhance the anti-tumor effect. Hence, bispecific antibodies (block PD-L1 and LAG3) may be a novel direction in future tumor treatment. LAG3 can also predict the therapeutic effect in tumors. LAG3 upregulation is strongly associated with the therapeutic effect of nivolumab in various mouse tumor models. Anti-LAG3 mAb clinical trials have certain limitations. First, the related mAbs are only used in animal trials, and there is a lack of sufficient clinical data. Second, due to the promising result of IMP321 in metastatic melanoma, the use of anti-LAG3 mAbs in metastatic tumors requires further study. Furthermore, the role of anti-LAG3 mAbs in early-stage tumors is unknown. Third, although there are no reports of anti-FGL1 mAbs undergoing clinical trials, the proven anti-tumor effect of FGL1 block indicates the enormous potential of developing anti-FGL1 mAbs. Fourth, irAEs need to be considered in combination immunotherapy, and enhancing the function of tumor-specific T cells without influencing other T cells may be critical to avoiding irAEs. Last, the combination of anti-LAG3 and anti-PD1 therapy receives the most attention, however, a better treatment effect may be achieved using a combination of other immune checkpoint inhibitors. Many studies have reported the predictive role of LAG3 in tumor prognosis. Given the inhibitory effect of LAG3 and FGL1 on the immune response, these oncogenes may predict a negative prognosis in several cancers. However, in some tumors, such as gastric cancer and melanoma, higher LAG3 expression indicates a better prognosis. However, further research is warranted to verify and clarify the underlying mechanisms. Moreover, predicting the role of LAG3 in breast cancer and pancreatic ductal adenocarcinoma prognosis is controversial, and we believe that the discrepancy between these two tumors can be attributed to tumor heterogeneity. Hence, more research is required to verify the predictive value of LAG3 in these cancer types. Furthermore, the expression of FGL1 only indicates a poor prognosis in lung cancer and gastric cancer, but its prognostic significance in other tumors is unclear and needs clarification **(** **Supplementary Table 2** **)**. LAG3 and FGL1 play significant roles in tumor therapy resistance. High FGL1 expression is associated with NSCLC EGFR-TKI treatment and gefitinib resistance, whereas low FGL1 expression is associated with sorafenib resistance in HCC. Furthermore, high LAG3 expression is related to anti-PD-1 therapy resistance. Thus, it is reasonable to consider that LAG3 and FGL1 may induce resistance to other immunotherapies or targeted therapies. The research potential of LAG3/FGL1 is enormous, understanding more about them may result in a broader implication, which may provide a novel strategy for tumor therapy.

## Author Contributions

A-PS and X-YT are mainly responsible for paper writing and typesetting. Y-LX, K-FZ, Y-JL, X-GS, and YL are mainly responsible for reference collection and table-form preparation. J-BZ, NM, and TJ are mainly responsible for paper review.

## Funding

National Natural Science Foundation of China, No. 82002421, Natural Science Basic Research Project of Shaanxi Province, No.2016JM8087, National Natural Science Foundation of China, No. 81001041.

## Conflict of Interest

The authors declare that the research was conducted in the absence of any commercial or financial relationships that could be construed as a potential conflict of interest.

## Publisher’s Note

All claims expressed in this article are solely those of the authors and do not necessarily represent those of their affiliated organizations, or those of the publisher, the editors and the reviewers. Any product that may be evaluated in this article, or claim that may be made by its manufacturer, is not guaranteed or endorsed by the publisher.

## Abbreviations

LAG3, Lymphocyte-activation gene 3; NK cells, natural killer cells; Treg cells, regulatory T cells; MHC, histocompatibility complex; LSECtin, lymph node sinusoidal endothelial cell C-type lectin; FREP1, fibrinogen-associated protein 1; KIRC, kidney renal clear cell carcinoma; NSCLC, non-small cell lung cancer; HCC, hepatocellular carcinoma; PCNSL, primary central nervous system lymphoma; DLBCL, diffuse large B-cell lymphoma; MIBC, muscle invasive bladder cancer; HNSCC, head and neck squamous cell carcinoma; IgSF, immunoglobulin superfamily; CCD, coil-coil domain; FD, fibrinogen-like domain; PD-1, programmed cell death protein 1; TIM3, T-cell immunoglobulin domain and mucin domain 3; CTLA-4, cytotoxic T lymphocyte-associated antigen-4; TNBC, triple negative breast cancer; APCs, antigen-presenting cells; ADAM, disintegrin and metalloproteinase domain-containing protein; mAbs, monoclonal antibodies; PFS, progression-free survival; irAEs, immune-related adverse effects.
